# *Trypoxylus dichotomus* Gut Bacteria Provides an Effective System for Bamboo Lignocellulose Degradation

**DOI:** 10.1128/spectrum.02147-22

**Published:** 2022-08-22

**Authors:** Junhao Huang, Linyao Weng, Xinqi Zhang, Kui Long, Xiaojiao An, Jinliang Bao, Hong Wu, Xudong Zhou, Shouke Zhang

**Affiliations:** a Department of Forestry Protection, School of Forestry and Biotechnology, Zhejiang A&F University, Hangzhou, China; b College of Chemistry and Materials Engineering, National Engineering & Technology Research Center for the Comprehensive Utilization of Wood-Based Resources, Zhejiang A&F University, Hangzhou, China; c Shanzhizhou Ecological Agriculture Company Limited, Pan’an, China; d State Key Laboratory of Subtropical Silviculture, Zhejiang A&F University, Hangzhou, China; Lerner Research Institute

**Keywords:** bamboo fiber, celluloytic activity, gut bacteria, metabolic pathway, rhinoceros beetle

## Abstract

Fast-growing bamboo may be a source of high-quality cellulose with the potential to contribute to energy sustainability, if an efficient and low-cost solution to bamboo cellulose decomposition can be developed. This study compared the gut microbiomes of rhinoceros beetle (*Trypoxylus dichotomus*) feeding on bamboo and wood fiber. The results revealed that diet has a distinctive effect on microbial composition in the midgut, including its most abundant microorganisms that in the fermentation and chemoheterotroph pathways. After identifying the 13 efficient bacterial isolates, we constructed a natural bacterial system based on the microbial relative abundance and an artificial bacterial system with equal proportions of each isolate to catabolize bamboo lignocellulose. The isolate Enterobacter sp. AZA_4_5 and the natural system showed higher degradation efficiency than other single strains or the artificial system. The results can thus serve as important reference for further research and development of a synthetic bacterial consortium to maximize lignocellulolytic ability.

**IMPORTANCE** Bamboo produces a great yield of lignocellulosic biomass due to its high efficiency in carbon fixing. The gut microbiome of *Trypoxylus dichotomus* differed between bamboo and wood fiber diets. The lignocellulosic pathways were enriched in the gut bacteria of the bamboo diet. The highly efficient bacterial isolates were identified from midgut, whereas the natural bacterial system as well as one isolate showed the higher degradation efficiency of bamboo lignocellulose. The results indicate that the gut bacteria could provide an effective system to utilize the bamboo lignocellulosic biomass.

## INTRODUCTION

Bamboo is a group of plants that includes some of the fastest-growing species in the world. It is widely distributed throughout the world, particularly Southeast Asia, the Americas, Africa, and Australia. Its rapid growth and tolerance to barren soil make bamboo an attractive choice for afforestation, carbon sequestration, and climate change mitigation (1, 2). In addition, the significant economic and cultural value of bamboo products has resulted in a continuous expansion of the industry across many parts of Asia (1, 2). However, in recent decades, a lack of sufficient management of bamboo plantations in southeastern China has led to its invasion into neighboring native forest, resulting in a loss of biodiversity (3, 4).

A potentially significant source of renewable carbon, fast-growing bamboo produces a high yield of lignocellulosic biomass (5, 6). However, the structural differences between bamboo and other types of lignocellulose make it challenging to develop a highly efficient and low-cost industrial process for bamboo lignocellulose degradation (5, 6). As a result, efforts are under way to integrate new technologies in the utilization of the vast bamboo fiber resources, increase their economic benefit, rekindle producer interest in bamboo, manage its spread, and ultimately secure the stability of native ecosystems (6, 7).

Consolidated bioprocessing is an effective approach to cellulose degradation (8, 9). Microbial bioenergy production utilizes a variety of microorganisms with complementary metabolic functions, such as the production of bioethanol, biobutanol, microbial lipids, and H_2_ (10). Numerous studies have been conducted to identify the cellulose-degrading bacteria that are the most effective in the relevant environment (8, 11–13). For instance, insects that feed on plant xylem have yielded certain cellulose-degrading microorganisms (12, 14). Among these, termites (Termitidae) (14), stag beetles (Lucanidae) (12), locusts (Orthoptera) (15), and cockroaches (Blattidae) (16) have all been investigated to isolate a series of gut microbial strains that contribute to cellulose degradation. However, until now, only the gut microbiota of certain herbivorous insects that feed on the fresh leaves or shoots of bamboo have been explored for their role in bamboo-cellulose degradation (7, 17). The study of gut bacteria and their lignocellulosic degradation functions remains a topic of interest due to their enormous potential in bio-based productions.

The rhinoceros beetle *Trypoxylus dichotomus* feeds on both wood and bamboo fibers and serves as an important decomposer. Like the stag beetle (Lucanidae) (12), *T. dichotomus* possesses a powerful gut microflora, which has been observed to ferment and decompose lignocellulose (18). It remains unclear whether *T. dichotomus* exhibits enrichment in bamboo-degrading gut bacteria, and whether there is a microbiome difference between individuals feeding on wood fiber or bamboo fiber. The current study used high-throughput sequencing of the 16S rRNA gene and its cDNA to compare the gut microbiomes of *T. dichotomus* larvae feeding on bamboo and wood fibers to determine the gut bacterial isolates specific to bamboo fiber. The differences in the metabolic pathways related to cellulose degradation were also compared between the two diets. Finally, the functional isolates that were significantly enriched in the guts of bamboo-fiber-feeding larvae were screened, and the cellulose-degrading ability of these isolates was evaluated in *in vitro* cultures. This study provides new avenues for the utilization of bamboo cellulose and contributes to developments in biomass utilization.

## RESULTS AND DISCUSSION

### Microbiome diversity and structural differences.

**FIG 1:**
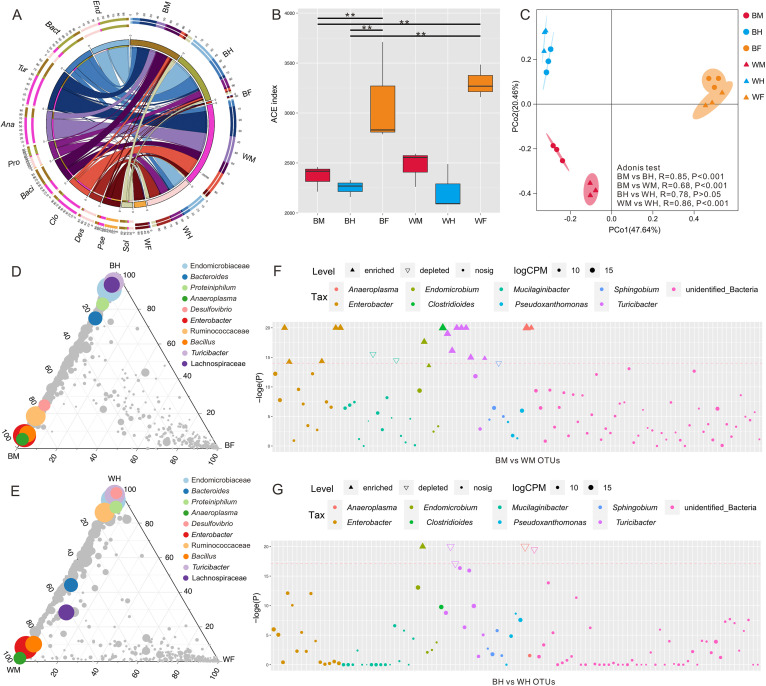
Comparison of microbial community structures based on 16S rRNA gene sequences. (A) Bacteria identified from the guts and food fibers, with the most abundant 10 taxa in genus level shown. End, *Candidatus_Endomicrobium*; Bact, *Bacteroides*; Tur, *Turicibacter*; Ana, *Anaeroplasma*; Pro, *Proteiniphilum*; Baci, *Bacillus*; Clo, *Clostridioides*; Des, *Desulfovibrio*; Pse, *Pseudoxanthomonas*; Sol, *Candidatus_Soleaferrea*. (B) ACE index analysis for the six groups. (C) Principal coordinates analysis plot of 16S rRNA genes weighted Bray-Curtis distances for the six groups (*P < *0.001, permutational multivariate analysis of variance [PERMANOVA] by Adonis). Ternary plot depicting all OTUs (>0.5%) in the gut samples of the bamboo fiber treatment groups (D) and the wood fiber treatment groups (E). Manhattan plots showing enriched OTUs in midgut (F) and hindgut (G) of bamboo-fiber-feeding beetles, with respect to wood-fiber-feeding beetles. Microbial DNA samples of guts and food: BM, bamboo-feeding midgut; BH, bamboo-feeding hindgut; BF, bamboo fiber; WM, wood-feeding midgut; WH, wood-feeding hindgut; WF, wood fiber.

All samples had saturated sparse curves, and the sequencing depth met the analytical requirements (Fig. S1 in the supplemental material). A total of 1,232,116 high-quality sequences were obtained from the 18 gut and food samples (68,450 reads on average per sample, ranging from 53,119 to 78,610 reads). Sequences were annotated to 45,322 operational taxonomic units (OTUs) with a 97% similarity level, belonging to 52 phyla and 699 genera. The dominant bacterial groups were Proteobacteria (301 OTUs), Firmicutes (286 OTUs) and Bacteroidetes (153 OTUs) (Fig. 1A; Fig. S2). Proteobacteria were most abundant in the diet samples (both bamboo and wood fibers), while Firmicutes were most abundant in the gut samples (Fig. 1A; Fig. S2). Proteobacteria are a dominant environmental bacterial community, including in the insect gut (19, 20), and their proportion in the diet of rhinoceros beetles is higher than their proportion in the beetles’ gut tract (Fig. 1A; Fig. S2). Firmicutes and Bacteroidetes usually exist in the rumen of animals and are known for the fermentation of polysaccharides (21, 22). Our results showed that Bacteroidetes were more abundant in the hindgut than in the midgut of the rhinoceros beetle (Fig. 1A; Fig. S2). They were positively correlated with the intake of lignocellulose (21). This is congruent with the fact that the hindgut of the third instar larva of *T. dichotomus* is recognized as a rumen due to its enlarged cavity, where solid food particles composed of cellulose, hemicellulose, pectin, and other polysaccharides are degraded (23).

The most abundant bacterial genera from the midgut of bamboo-fiber-feeding rhinoceros beetles are *Turicibacter* (6.26%), *Bacillus* (5.80%), and *Anaeroplasma* (3.39%), whereas wood-fiber-feeding individuals showed an abundance of *Turicibacter* (9.64%), *Anaeroplasma* (8.55%), and *Clostridioides* (7.04%) (Fig. 1A). *Candidatus_Endomicrobium* (9.07%, 7.03%) and *Bacteroides* (6.64%, 2.71%) were the dominant bacterial genera in the hindgut of rhinoceros beetles feeding on bamboo and wood (Fig. 1A). Lignocellulosic degradation often requires close cooperation between the midgut, hindgut, and their respective microbiomes to complete the complex digestion process (12). Metagenomic and meta-transcriptome studies have shown that *Turicibacter* harbors a variety of hydrolytic enzymes, including glycoside hydrolytic enzymes, which can catalyze the hydrolysis of lignocellulose (24, 25). *Candidatus_Endomicrobium* and *Bacteroides* can further decompose and digest the remaining cellulose in the hindgut (26). The ACE diversity index revealed that the bacterial diversity in both diet types was significantly higher than in the gut samples (*P* < 0.05), while the dietary differences did not have an effect on diversity in the midgut and hindgut (Fig. 1B, Table S2). In addition, the Chao1, Shannon-Wiener, and Simpson indices revealed a significantly higher diversity in the bamboo fiber diet than in the guts of the bamboo-fiber-feeding larvae. The results differed from those obtained from the wood-fiber-feeding larvae (Table S2). Usually, the gut microbiomes of herbivores are susceptible to environmental changes (27). However, this study shows that the bamboo and wood fiber diet types had little effect on microbial diversity in the midgut and hindgut, even though the microbial diversity in the diets was significantly higher than that in the larval gut. Although the diet was considered a major shaping factor of insect gut microbiomes (17), these findings indicate that the gut microbiomes of *T. dichotomus* larvae may maintain a certain stability on dietary interference.

Pairwise analysis showed a significant difference (*P < *0.05) between the midgut microbiomes feeding on bamboo and wood fibers, whereas no significant difference was found between the hindgut microbiomes of the larvae feeding the two diet types (Fig. 1C, Table S3). The PCoA result showed that the samples were distinctively grouped by diet, midgut, and hindgut (Fig. 1C). Compared to the differences from diets and hindguts, the structure of the midgut microbiomes remained relatively stable whether a mono diet of wood or bamboo fiber was consumed. This may be related to the low content of plant secondary metabolites in wood and bamboo fibers (27–29), which are often toxic, acidic, or alkaline, and influence the diversity of the gut microbiome (27, 30). Polysaccharides are generally not toxic to gut bacteria, further explaining the low degree of variability in the microbiome, which subsists on different sources of cellulose in different parts of the gut.

Ternary plots were used to compare the dominant microbial OTUs in the midgut and hindgut between the two fiber diets (Fig. 1D and E). The result indicates that the microbiomes in the gut and diet fibers did not share many common OTUs, while the midgut and hindgut microbiomes from each diet showed a higher degree of similarity. Three OTUs of Enterobacter, *Anaeroplasma*, and *Bacillus* were significantly enriched in the midguts of larvae on both diets, while another three OTUs of Endomicrobiaceae, *Turicibacter*, and *Proteiniphilum* were enriched in the hindguts of both diet groups. Enriched in the hindgut of the bamboo-fiber-feeding group, two OTUs (Lachnospiraceae and *Bacteroides*) became common bacteria in the midgut and hindgut of wood-fiber-feeding individuals. Meanwhile, one OTU of *Desulfovibrio* was observed to be enriched in the midgut of the bamboo diet group rather than in the hindgut of the wood diet group. A Manhattan plot was used to further demonstrate the variation in the microbial structure in the midgut and hindgut of the rhinoceros beetle on the two diets (bamboo fiber versus wood fiber) (Fig. 1F and G). After the elimination of low abundant OTUs, 3 depleted OTUs were found in the midgut of larvae feeding on bamboo compared with those feeding on wood fiber: *Mucilaginibacter* (2) and *Sphingobium* (1); and 17 enriched OTUs: Enterobacter (5), *Endomicrobium* (2), *Clostridioides* (1), *Turicibacter* (7), and *Anaeroplasma* (2). However, the difference in hindgut microbial structure was significantly decreased in the larvae on the two diets, where only 4 depleted OTUs, *Turicibacter* (2) and *Anaeroplasma* (2), and 1 enriched OTU, *Endobiomicrobium* (1), were detected. The results showed that the midgut microbiome was significantly enriched in the larvae feeding on bamboo rather than wood fiber, with Enterobacter and *Turicibacter* as the most enriched genera. Previous studies have shown that the genus Enterobacter contains a variety of bacterial species capable of degrading cellulose in various environments, including bovine stomachs (31), soil (32), and insect guts (33, 34). *Turicibacter* is predominantly found in the fermentation process of composting (8, 29); however, its role in the degradation of lignocellulose has not yet been determined.

### Comparison of functional differences in the gut microbiome.

After the coverage of sequencing depth met the analysis requirements (Fig. S3), we analyzed microbiome diversity and the functional differences between the four groups of gut microbial cDNA samples. Revealed by the four alpha diversity indices at the transcriptome level, the significant differences (*P < *0.05) among the guts from the different diet groups largely varied (Table S4). However, consistent with the results of 16S rRNA gene sequencing, no significant differences were found in the diversity and structure of the hindgut microbiome of the two diet groups (Table S4, S5), which were clustered distantly from the midgut microbiomes (Fig. S4).

**FIG 2:**
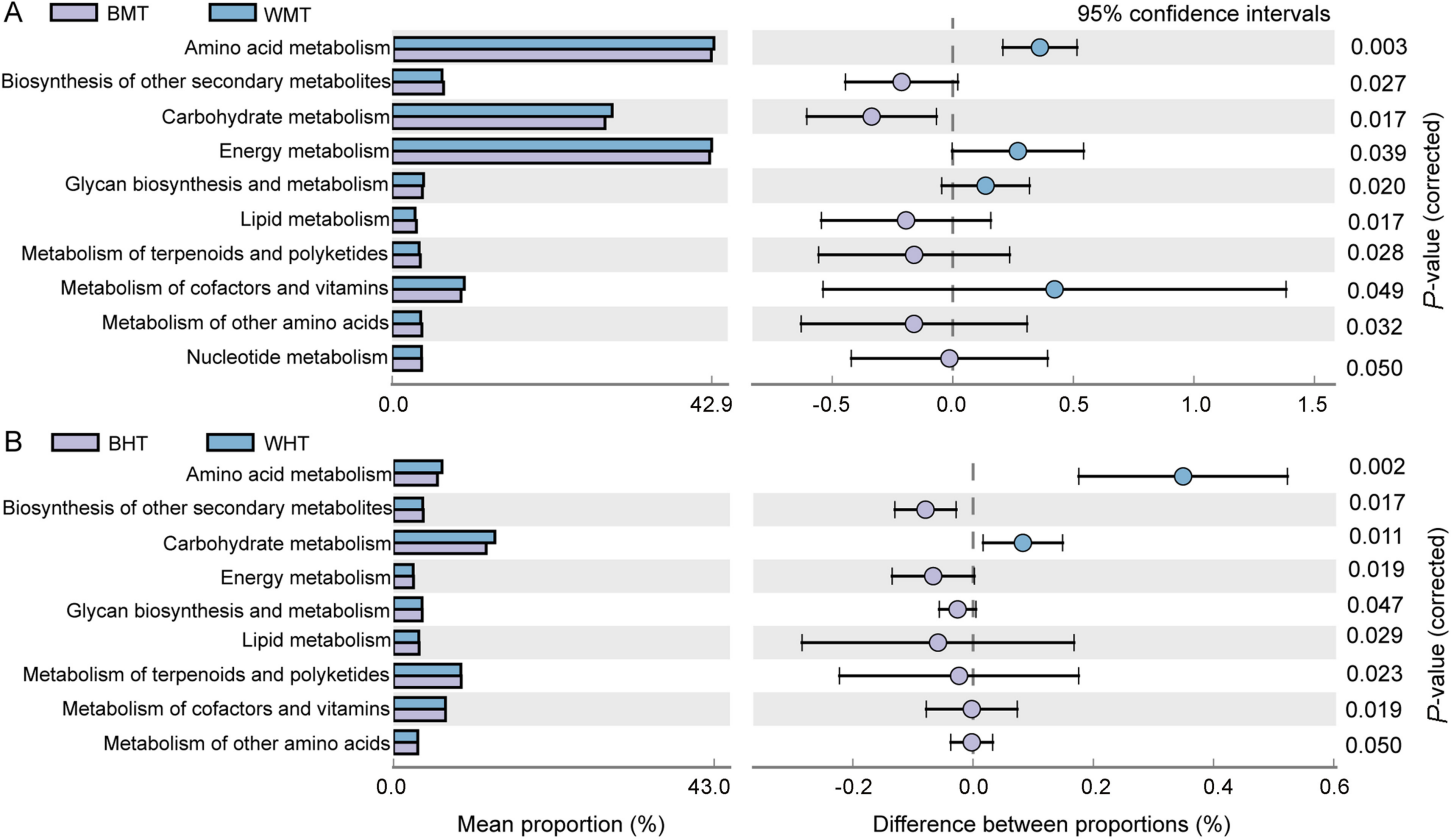
PICRUSt2-identified functional differences in the gut microbiome based on cDNA of the 16S rRNA gene, assessed using Welch's *t* test. (A) Midgut differences. (B) Hindgut differences. The different functions are shown along the ordinate, while mean abundance is shown along the abscissa. The differences in abundance among the functions along the *x* axis. Dot colors indicate the species in which the corresponding function is more abundant; error bars show the 95% confidence interval of the difference between two diets. The significance levels of differences among functions are shown along the *y* axis. Microbial cDNA samples of guts: BMT, bamboo-feeding midgut; WMT, wood-feeding midgut; BHT, bamboo-feeding hindgut; WHT, wood-feeding hindgut.

PICRUSt2 identified functional differences between the gut microbiomes of the two diet groups (Fig. 2). Among the top 30 significantly enriched KEGG pathways, those related to nutrient metabolism and with significant differences between the midgut and hindgut were selected for comparison. The result showed that the difference in diet type did not have a drastic effect on midgut metabolism (Fig. 2A). In the midgut of bamboo-fiber-feeding larvae, metabolism pathways of amino acid, carbohydrate, and energy were the most abundant, and biosynthesis of other secondary metabolites, lipid and terpenoid metabolism, and polyketides and other amino acid pathways were also significantly enriched (Fig. 2A). Although the pathways of amino acid, carbohydrate, and energy metabolisms were significantly enriched in the midgut, they decreased in the hindgut, with carbohydrate metabolism possibly representing the most active metabolic pathway (Fig. 2A and B). A previous study has reconstructed the metabolic pathways of 737 bacteria, archaea, and fungi with high-quality metagenome-assembled genomes, and determined that the cross-domain partnership between fungi and methanogens could produce acetic acid, formic acid, and methane, while bacteria-dominated flora mainly produced short-chain fatty acids, including propionic acid and butyric acid (13). Our results showed that a similar degradation process occurred in the intestine after the intake of bamboo fibers. Gut bacteria may thus play an important role in beetles’ ability to use lignocellulose for their own energy supply.

**FIG 3:**
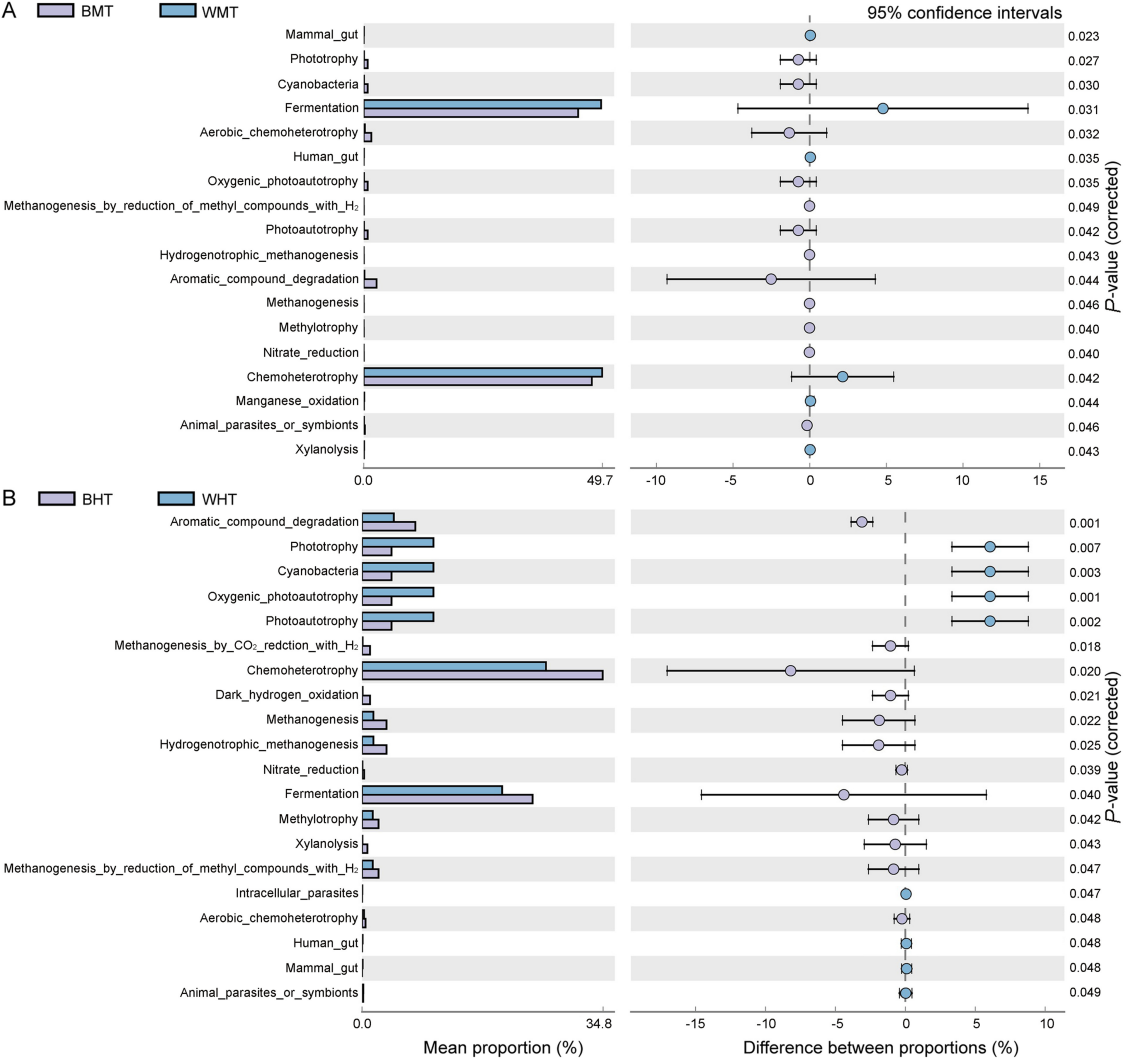
FAPROTAX-identified functional differences in the gut microbiome based on cDNA, assessed using Welch's *t* test. (A) Functional differences between BMT and WMT. (B) Functional differences between BHT and WHT. The different functions are shown along the ordinate, while mean abundance is shown along the abscissa. The differences in abundance among the functions along the *x* axis. Dot colors indicate the species in which the corresponding function is more abundant; error bars show 95% confidence interval of the difference between two groups The significance levels of differences among functions are shown along the *y* axis. Microbial cDNA samples of guts: BMT, bamboo-feeding midgut; WMT, wood-feeding midgut; BHT, bamboo-feeding hindgut; WHT, wood-feeding hindgut.

Functional annotation by FAPROTAX was conducted to predict the biochemical cycling process in the intestinal tract, in particular the cycling of nutrient elements such as carbon, hydrogen, and nitrogen (35). Eighteen different functional pathways were observed in the larval midguts after feeding on the two fiber diets. The most abundant microorganisms were those in the fermentation and chemoheterotroph pathways, which showed significantly higher abundance in the midgut microbiomes of wood-feeding larvae (*P < *0.05) (Fig. 3A). In addition, 20 significantly different functions were noted in the hindguts of the larvae feeding on the two fiber diets. Among these, the pathways of aromatic compound degradation, methanogenesis by CO_2_ reduction with H_2_, chemoheterotrophy, dark hydrogen oxidation, methanogenesis, hydrogenotrophic methanogenesis, nitrate reduction, fermentation, methylotrophy, and aerobic chemoheterotroph and methanogenesis by reduction of methyl compounds with H_2_ showed significantly higher abundance (*P < *0.05) in the wood-fiber-feeding group (Fig. 3B). Ceja-Navarro et al. (2019) demonstrated that lignocellulosic decomposition and fermentation occur sequentially in different compartments. The distinguishing characteristics of the midgut and hindgut facilitate the selection of the microbiome and its metabolic pathways. Metaproteogenomics results showed that a higher oxygen concentration in the midgut promotes lignocellulosic depolymerization, while a thicker gut wall in the hindgut reduces oxygen diffusion, facilitates hydrogen accumulation, and promotes fermentation, homologous acetylation, and nitrogen fixation (12). Therefore, we believe that during the process of the development and utilization of lignocellulolytic bacterial flora in insect gut microbiomes, the difference in oxygen content between midgut and hindgut has an effect on the structure and function of the flora, impacting the degradation efficiency of lignocellulose.

### Isolation of functional gut bacteria for bamboo fiber degradation.

**FIG 4:**
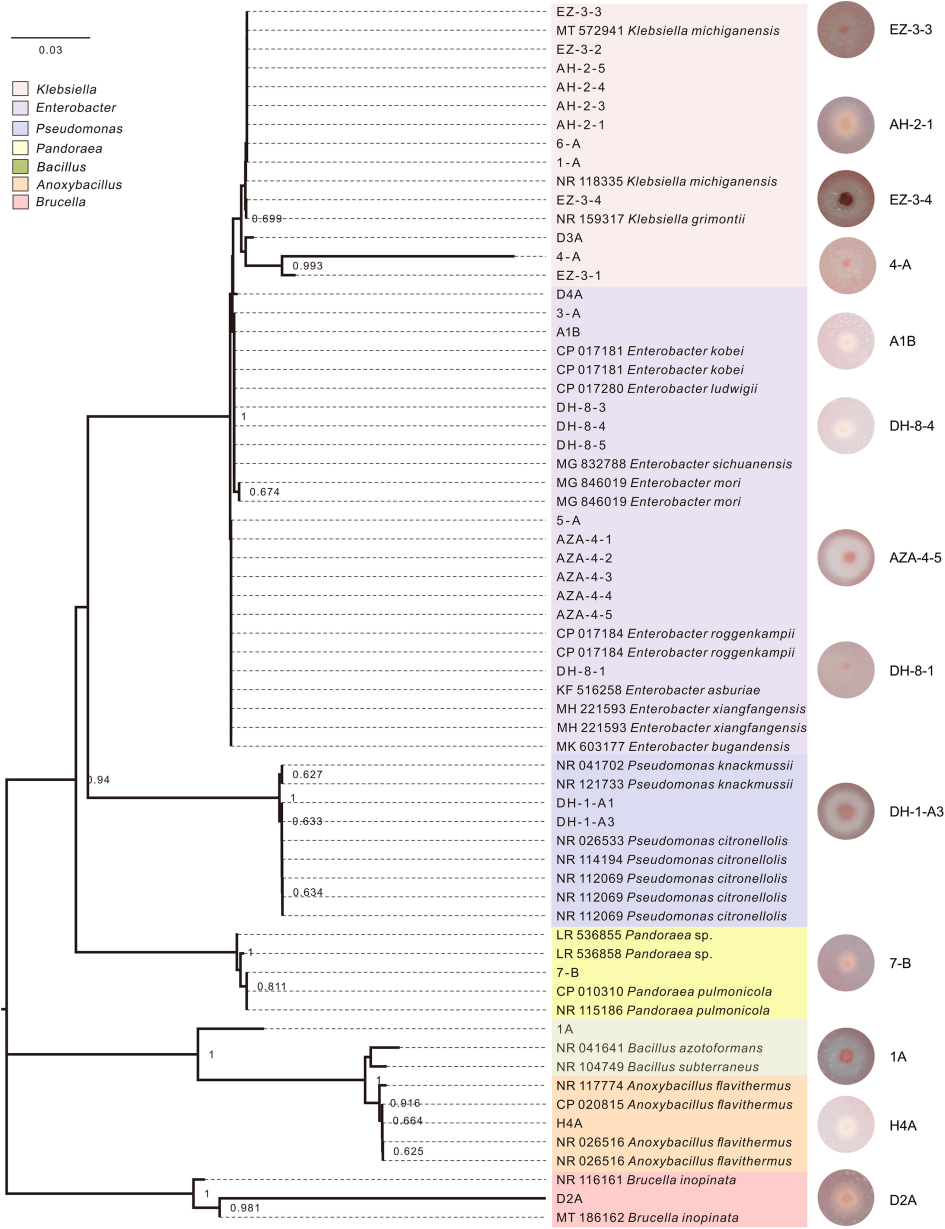
Maximum likelihood phylogenetic tree of 16S rRNA gene sequences of cellulolytic bacteria isolates selected from CMC-Na single carbon source medium. The tree included a total of 31 cellulolytic bacterial isolates screened and identified from the midgut and hindgut of bamboo-fiber-feeding larvae, as well as 34 closely related sequences obtained from NCBI. The plates on the right showed the 13 isolates with higher degradation efficiency.

Early studies on lignocellulolytic bacteria suggested that it was necessary to find, develop, and utilize single bacterial species (36). However, recent work has revealed that a multistrain compatible microbial system can achieve a higher lignocellulosic degradation efficiency (37). In our study, a total of 31 cellulolytic bacterial isolates were screened and identified from the midgut and hindgut of bamboo-fiber-feeding larvae. They belonged to seven genera, including Klebsiella, Enterobacter, Pseudomonas, *Pandoraea*, *Bacillus*, *Anoxybacillus*, and *Thermoflavimicrobium* (Fig. 4). Enterobacter is capable of cellulose degradation under both aerobic and anaerobic conditions (38). Together with Pseudomonas, Enterobacter appeared in the top 20 strains for degrading plant cell walls (Dar et al., 2018, [39]). Klebsiella has been reported to produce cellulase with degradation ability in the gut of Helicoverpa armigera (Noctuidae) (40). *Anoxybacillus* is a vegetative, anaerobic, thermophilic bacterium that metabolizes various sugars and uses crystalline cellulose as a carbon source (41). *Pandoraea* is a common bacterial component of soil environments (42), which is known for its alkaline tolerance and potential for degrading crude fibers (43).

**FIG 5:**
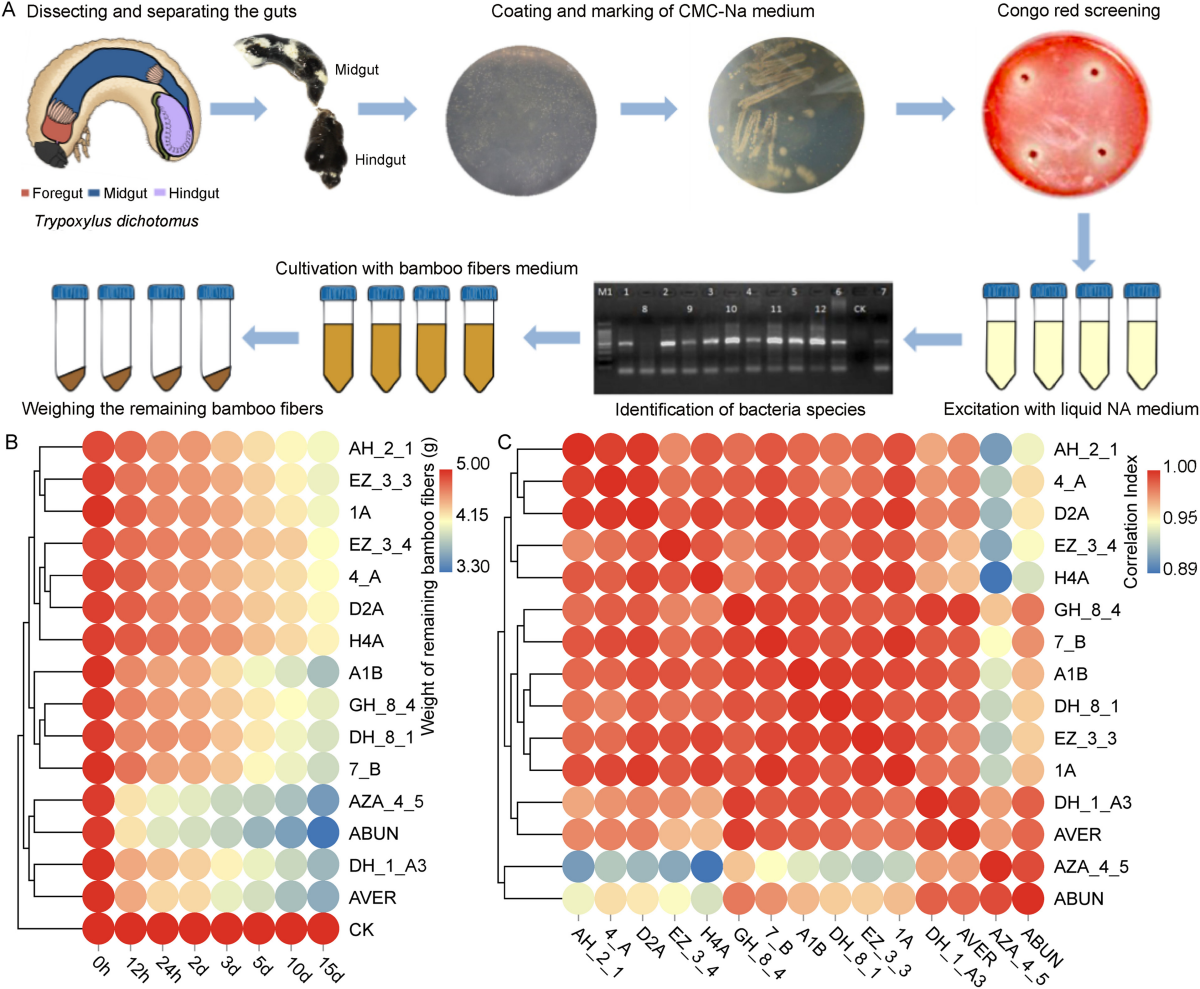
Degradation efficiency analysis of bamboo-fiber-degrading bacteria isolated from larval gut. (A) Isolation, purification, and functional validation of gut lignocellulosic degrading bacteria. (B) Analysis of degradation efficiency of bamboo fibers by single and mixed bacteria systems. (C) Analysis of the relationship between the degradation efficiency of bamboo fiber by single bacteria and mixed bacteria.

Thirteen bacterial isolates from seven genera (*Anoxybacillus* sp. H4A; *Bacillus* sp. D2A; *Thermoflavimicrobium* sp. 1A; Pseudomonas sp. DH-1-A3, from hindgut; Enterobacter spp. A1B and AZA-4-5 from midgut, DH-8-1 and DH-8-4 from hindgut; Klebsiella spp. EZ-3-3 and EZ-3-4 from midgut, 4-A and AH-2-1 from hindgut; *Pandoraea* sp. 7-B from midgut) were determined to be efficient cellulolytic bacteria due to the larger diameter of their hydrolytic circles (Table S6). They were further cultured to assess their cellulolytic efficiency in bamboo fiber degradation (Fig. 5B). We found that the dynamic trends of bamboo fiber degradation displayed by Enterobacter sp. AZA_4_5, Pseudomonas sp. DH_1_A3, the natural bacterial system, and the artificial bacterial system were largely consistent with each other, and their degradation efficiency was significantly higher than that of other groups (Fig. 5B). In addition, the degradation efficiency of the artificial bacterial system showed the strongest correlation with Enterobacter sp. AZA_4_5, while the natural bacterial system was closely correlated with Pseudomonas sp. DH_1_A3 (Fig. 5B and C). The results indicated that all the bacteria cultures displayed a significant degradation efficiency in 12 h, while Enterobacter sp. AZA_4_5 and the natural bacterial system showed significant higher efficiency than the other bacteria in most time periods (Table S8). The differences in the degradation capacities of these two isolates may be due to their different oxygen requirements in the midgut and hindgut (12). Enterobacter sp. AZA_4_5 may be able to take advantage of oxygen in the rapid oxidation of lignocellulose at the beginning of fermentation, while Pseudomonas sp. DH_1_A3 requires a low oxygen environment to play its role. Despite this, the natural bacterial system showed higher fiber degradation efficiency (Fig. 5B) than the synthetic bacterial communities reported in other studies (44, 45).

### Conclusions.

Comparing bamboo-fiber-feeding and wood-fiber-feeding larvae of *T. dichotomus*, we found differences in the composition, structure, and function of their gut microbiome, which were related to the structural characteristics and microbiome inconsistency of the midgut and hindgut of the larvae. Thirteen isolates screened from the gut of bamboo-fiber-feeding larvae showed higher lignocellulosic degradation functions, while the artificial microbial system constructed based on bacterial relative abundance demonstrated the highest lignocellulosic degradation ability. We can conclude that high-throughput sequencing (HTS) technology can serve as an important basis for constructing synthetic bacterial communities to develop highly efficient bamboo fiber degradation schemes.

## MATERIALS AND METHODS

### Sample collection.

The *T. dichotomus* beetles were reared at Pan’an, Zhejiang Province, China (latitude: 29°15'48” N, longitude: 120°25'48” E; altitude: 60 m) on bamboo and wood fibers (sawdust), respectively, as two diet groups, i.e., a bamboo-fiber-feeding group and a wood-fiber-feeding group. The larvae were raised at room temperature in the laboratory until the 3rd instar. Subsequently, 15 healthy 3rd-instar larvae (each weighing 27.3 g on average) were selected from each diet group. After starvation for 24 h, their midguts and hindguts were separated in a phosphoric acid buffer (pH = 7.2) using sterile instruments and techniques for microbial examination (17, 46). The guts from five individuals were pooled as a single sample, resulting in three samples for each gut group (bamboo-fiber-feeding: BM, bamboo midgut; BH, bamboo hindgut; wood-fiber-feeding: WM, wood midgut; WH, wood hindgut) (Table S1). Meanwhile, three samples (each weighing 15 g) of each diet type (wood and bamboo fibers) were randomly collected (BF, bamboo fiber; WF, wood fiber) to test the microbiome in the food. In total, 18 samples (2 diets × 3 guts and food × 3 replicates) were prepared for DNA extraction. In addition, another 12 samples of the four gut groups were prepared for RNA extraction and microbial cDNA sequencing to form the new groups (BMT, BHT, WMT, and WHT). All samples were stored at –80°C until use.

### Microbial DNA/RNA extraction, PCR amplification, and sequencing.

The 18 samples of guts and diets were homogenized and applied for DNA extraction, using the QIAamp Fast DNA Stool minikit (Qiagen, Germany) following the manufacturer’s instructions. The additional 12 gut samples were homogenized for microbial RNA extraction, using the TRIzol method described by Kang et al. (2009) (47). RNA degradation and contamination were monitored on 1% agarose gel. RNA purity was checked with the NanoPhotometer spectrophotometer (IMPLEN, CA, USA). RNA integrity was assessed using the RNA Nano 6000 assay kit of the Bioanalyzer 2100 system (Agilent Technologies, CA, USA). cDNA libraries were generated using the NEBNext UltraTM RNA Library Prep Kit for Illumina (NEB, USA), according to the manufacturer’s recommendations.

The target region of the 16S rRNA genes of the above 30 DNA samples was amplified using the primers 341F (5′-CCTAYGGGRBGCASCAG-3′) and 806R (5′-GGACTACNNGGGTATCTAAT-3′) (27). PCR conditions were as follows: 94°C for 2 min, followed by 30 cycles at 98°C for 10 s, 62°C for 30 s, and 68°C for 30 s with a final extension at 68°C for 5 min. The Ion Plus Fragment Library Kit 48 RXNS Kit (Thermofisher, USA) was used for library construction. After Qubit detection and quantification, all the libraries were sequenced on an Illumina Novaseq 6000 PE250 platform.

### Data processing and bioinformatics analysis.

Raw paired-end reads of the 16S rRNA gene and its cDNA sequences obtained in this study were deposited in the NCBI database under the accession numbers PRJNA781432 and PRJNA781626. To obtain high-quality clean reads, the raw reads were filtered using FASTP (version 0.18.0) (48) to remove those reads containing >10% of unknown nucleotides, and those where <50% of all bases had quality values >20. Paired-end clean reads were merged as raw tags using FLASH (version 1.2.11), with a minimum overlap of 10 bp and a mismatch error rate of 2% (49). Noisy sequences were removed using the QIIME2 pipeline as follows: first, low-quality regions (default minimum quality ≤3; default minimum length ≥3) in the raw tags were identified, and the raw tag was split at the first low-quality base in the region; next, we removed tags where the length of the continuous sequence of high-quality bases was <75% of the tag length. Clean tags were searched against the reference database (version r20110519) using the UCHIME algorithm (50) to perform reference-based chimera checking. All chimeric tags were removed. The remaining effective tags were used for further analysis. The effective tags were clustered into OTUs at ≥97% similarity using the UPARSE (version 9.2.64) pipeline. The tag sequence with the highest abundance within each cluster was selected as the representative sequence. The representative sequences were then taxonomically identified with a naive Bayesian model using the RDP classifier (version 2.2) against the SILVA database (version 138) (51), with a confidence threshold of 0.8 (52).

### Gut microbial species diversity.

Calculations and plots were mainly carried out using R (version 4.0.3) (53). OTU rarefaction, rank abundance curves, and stacked bar plots of community composition were visualized using the R package “ggplot2” (version 3.3.3) (54). An UpSet plot was drawn using the R package “UpSetR” (version 1.4.0) (55) to identify unique and shared OTUs. Circular layout representations of species abundance were graphed using Circos (version 0.69-3) (54). Circular plot was generated using a dynamic real-time interactive online platform (http://www.omicsmart.com). Ternary plots of species abundance were constructed using the R package “ggtern” (version 3.3.5) (56). Manhattan plots were performed on the Tutools platform (http://www.cloudtutu.com) to assess the enrichment of OTUs between midgut and hindgut.

Alpha diversity indices (including ACE, Chao1, Shannon-Wiener, and Simpson) were calculated using QIIME2. Indices of alpha diversity and functional differences from KEGG pathways between groups were compared using Welch’s *t* test and the Wilcoxon rank test in the R package “vegan” (version 2.5-7) (57). The Bray-Curtis and Jaccard distance matrices were calculated using the R package “vegan” (57) to compare the differences between the samples. Multivariate statistical analyses of the (un-)weighted UniFrac, Jaccard, and Bray-Curtis distances, including principal coordinates analysis (PCoA), were calculated using the R package “vegan” (57) and plotted using the package “ggplot2” (version 2.2.1). Permutational multivariate analysis of variance (PERMANOVA) was performed by the R package “vegan” to compare the significant differences among the community composition of gut bacteria. The KEGG pathways associated with the OTUs were identified using PICRUSt2 (version 2.1.4) (58) and FAPROTAX (Functional Annotation of Prokaryotic Taxa) (35).

### Isolation of cellulolytic bacteria.

To screen the cellulolytic bacteria from the midgut of the third instar larvae, samples were diluted gradiently and spread on solid media with carboxymethylcellulose-Na (CMC-Na) as a single carbon source. CMC-Na solid medium includes 1.9 g/L K_2_HPO_4_, 0.94 g/L KH_2_PO_4_, 1.6 g/L KCl, 1.43 g/L NaCl, 0.15 g/L NH_4_Cl, 0.037 g/L Mg_2_SO_4_·7H_2_O, 0.017 g/L CaCl_2_·2H_2_O, 10.00 g/L CMC-Na, and 16.00 g/L agarose, with pH = 7.2. The medium was sterilized at 120°C for 20 min. Three midgut contents of larvae feeding on bamboo fibers were mixed with 30 mL phosphate buffer in a sterilized 50-mL centrifuge tube. After thorough mixing, the solution was centrifuged at a low speed at 4°C for 5 min, then 10 mL of suspension was collected for bacterial isolation. Ten μL suspension was used for a concentration dilution of 10^−1^ to 10^−7^. The diluted suspension was spread on the CMC-Na solid medium for coating. Five replicates were prepared for each concentration. The bacteria were cultured in an incubator at 37°C and observed every 8 h. After 24 h, isolates were purified using streaking method. After another 24 h, purified individual isolates were further selected for coating on CMC-Na solid medium. By Congo red staining method, the bacterial isolates with hydrolytic circles were preliminarily screened as potential cellulolytic bacteria.

These isolates were then cultured for rejuvenation in NA liquid medium (5.00 g/L peptone, 3.00 g/L beef extract, 5.00 g/L NaCl, pH = 7.2) at 37°C and 200 rpm on a shaking table. The turbidity of the bacterial culture was observed every 12 h. After the bacterial liquid became turbid, the culture was coated on CMC-Na solid medium with Congo red for rescreening. The isolates with a distinct hydrolytic circle were finally selected as cellulolytic bacteria and cultured in NA medium for population expansion at 37°C and 200 rpm for 24 h. Then 2 mL of culture were used for bacterial DNA extraction by QIAamp Fast DNA Stool minikit (Qiagen, Germany). 16S rRNA gene was amplified using primers 8F (5′-TTTGATCCTGGCTCAG-3′) and 926R (5′-CCGTCAATTCCTTTAAGTTT-3′) and sequenced on the ABI_3730 platform. The obtained sequences were compared to those in the NCBI database, and the phylogenetic analysis was conducted for species identification.

### Degradation of bamboo fiber by single bacterial isolate and systems.

Among all the cellulolytic bacterial isolates obtained from the midgut and hindgut of bamboo-fiber-feeding larvae, 13 isolates showed higher degradation efficiency. Activated cultures of these 13 individual isolates and their mixed bacterial systems (in NA medium, OD = 0.54, 600 nm) were prepared to verify their degradation efficiency for bamboo fiber. The artificial bacterial system (AVER) was prepared with equal proportions (385 μL of each isolate), while the natural bacterial system (ABUN) was mixed according to the relative abundance of each isolate revealed in HTS results (250 μL H4A, 150 μL D2A, 475 μL 1A, 500 μL A1B, 500 μL AZA_4_5, 500 μL DH_8_1, 500 μL DH_8_4, 250 μL EZ_3_3, 250 μL EZ_3_4, 250 μL 4_A, 250 μL AH_2_1, 525 μL 7_B, 600 μL DH_1_A3) (Table S7). Five mL culture of each isolate and mixed bacterial systems were inoculated into 30 mL liquid medium (1.9 g/L K_2_HPO_4_, 0.94 g/L KH_2_PO_4_, 1.6 g/L KCl, 1.43 g/L NaCl, 0.15 g/L NH_4_Cl, 0.037 g/L Mg_2_SO_4_·7H_2_O, 0.017 g/L CaCl_2_·2H_2_O) in a 50-mL centrifuge tube, respectively, each added with 0.15 g bamboo fiber (dry weight, powder) as a single carbon source. Meanwhile, 5 mL sterile water was added into 30 mL medium as control. Thirty-five tubes were prepared for each culture (37°C, 200 rpm). Turbidity of the bacterial culture was observed every 12 h. Five tubes (replicates) of each culture were collected at seven fixed intervals (12 h, 24 h, 2 days, 3 days, 5 days, 10 days, and 15 days). The tubes were then centrifuged at high speed, and supernatant was removed. The dry weight of precipitate (remaining bamboo fiber) in each tube was recorded.

Heatmap plots were generated using dynamic real-time interactive online platform Omicsmart (http://www.omicsmart.com). Remaining weight of bamboo fibers was compared using one-way ANOVA analysis and Tukey's honestly significant difference (HSD) test in the R packages “car” (version 3.0) (59) and “multcomp” (version 1.4-10) (60) for testing the digestibility of the bacterial cultures.

### Data availability.

Raw sequences obtained in this study were deposited in the National Center for Biotechnology Information (NCBI) database under the accession numbers PRJNA781432 and PRJNA781626 (for SRA data), and OP056577–OP056606 and OP060800 (for 31 bacterial isolates).
