# Supplementary material for: Trypoxylus dichotomus Gut Bacteria Provides an Effective System for Bamboo Lignocellulose Degradation
Source: Microbiol Spectr. 2022 Aug 22;10(5):e02147-22. doi: 10.1128/spectrum.02147-22 (PMC9602259; doi:10.1128/spectrum.02147-22)
Supplement: Supplemental file 1 — Supplemental material. Download spectrum.02147-22-s0001.pdf, PDF file, 0.6 MB [file spectrum.02147-22-s0001.pdf]

Trypoxylus dichotomus gut bacteria provides an effective system for bamboo lignocellulose degradation

Junhao Huang [a,*], Linyao Weng [a], Xinqi Zhang [a], Kui Long [a], Xiaojiao An [b], Jinliang Bao [c], Hong Wu [a], Xudong Zhou [a,d], Shouke Zhang [a,d,*]

[a] Department of Forestry Protection, School of Forestry and Biotechnology, Zhejiang A&F University, Hangzhou 311300, China

[b] College of Chemistry and Materials Engineering, National Engineering & Technology Research Center for the Comprehensive Utilization of Wood-Based Resources, Zhejiang A&F University, Hangzhou 311300, China

[c] Shanzhizhou Ecological Agriculture Company Limited, Pan'an 322300, China

[d] State Key Laboratory of Subtropical Silviculture, Zhejiang A&F University, Hangzhou 311300, China

*__Correspondence to__: Junhao Huang and Shouke Zhang, School of Forestry and Biotechnology, Zhejiang A&F University, Hangzhou 311300, China. Tel: 86-571-63732758; fax: 86-571-63740809; email: huangjh@zafu.edu.cn and socrazhang@zafu.edu.cn

Fig. S1 Bacterial rarefaction curves were used to assess the depth of coverage for each sample of 16S rRNA gene. Each sample was distinguished by different line colors.

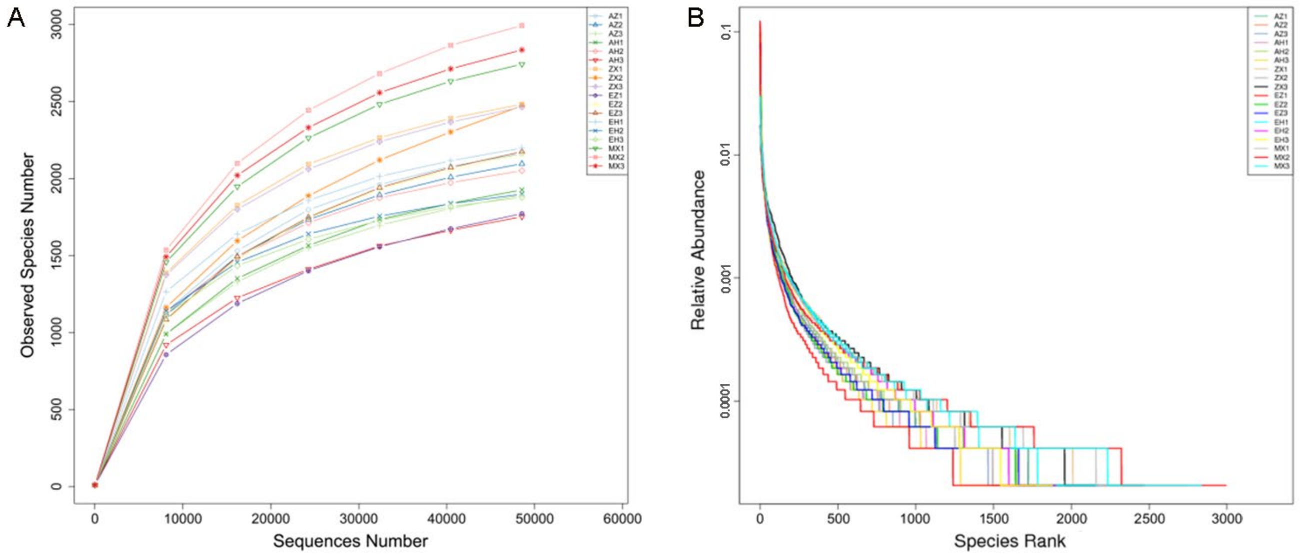

Fig. S2 UPGMA clustering tree based on weighted_unifrac distance. The relative abundances of the top ten phyla in all samples are indicated; the rest of phyla are indicated as ‘Others’. Microbial DNA samples of guts and food: BM, bamboo-feeding midgut; BH, bamboo-feeding hindgut; BF, bamboo fiber; WM, wood-feeding midgut; WH, wood-feeding hindgut; WF, wood fiber.

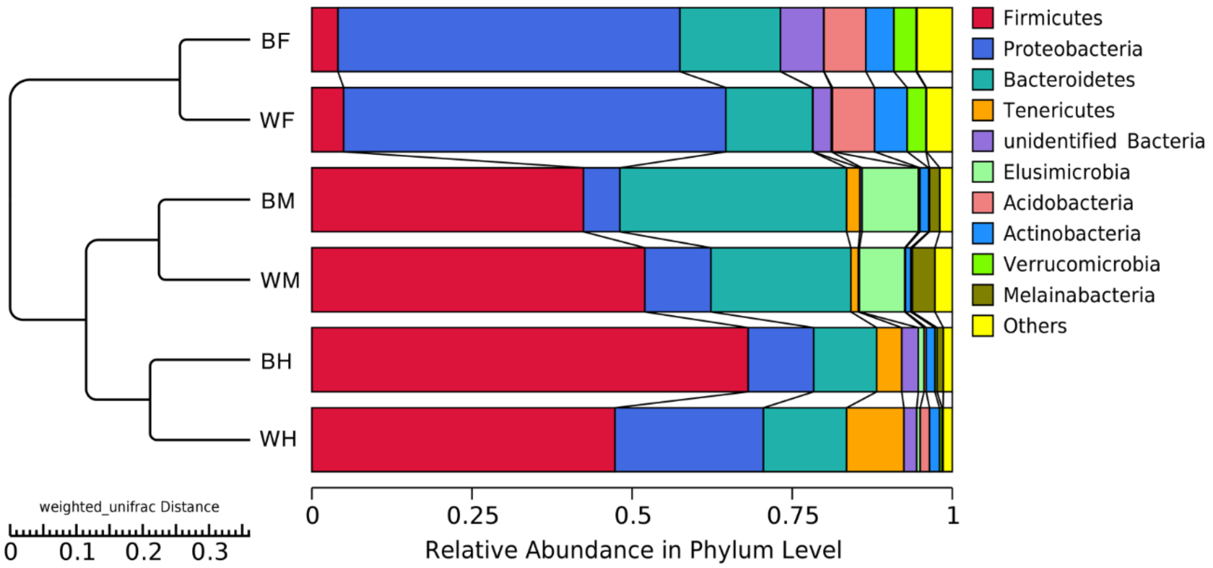

Fig. S3 Bacterial rarefaction curves were used to assess the depth of coverage for each cDNA sample of 16S rRNA gene. Each sample was distinguished by different line colors.

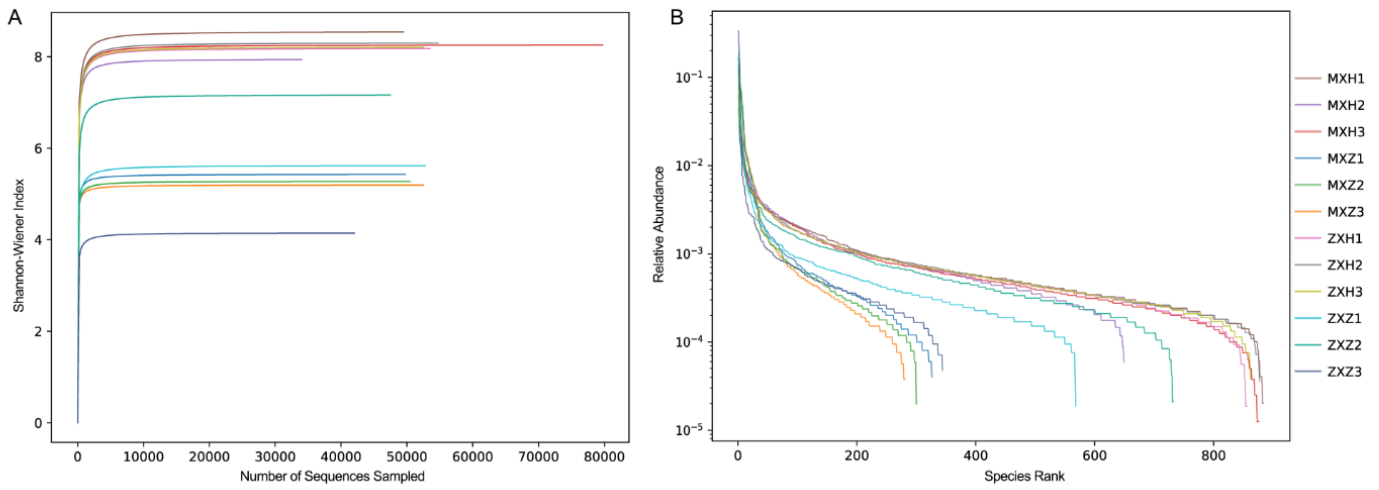

Fig. S4 Diversity and structures of gut microbiome based on cDNA of 16S rRNA gene. (A) Analysis of Shannon-Wiener diversity. (B) Principal coordinates analysis based on weighted Bray_Curtis distances by Anosim ($P < 0.001$) Microbial cDNA samples of guts: BMT, bamboo-feeding midgut; WMT, wood-feeding midgut; BHT, bamboo-feeding hindgut; WHT, wood-feeding hindgut.

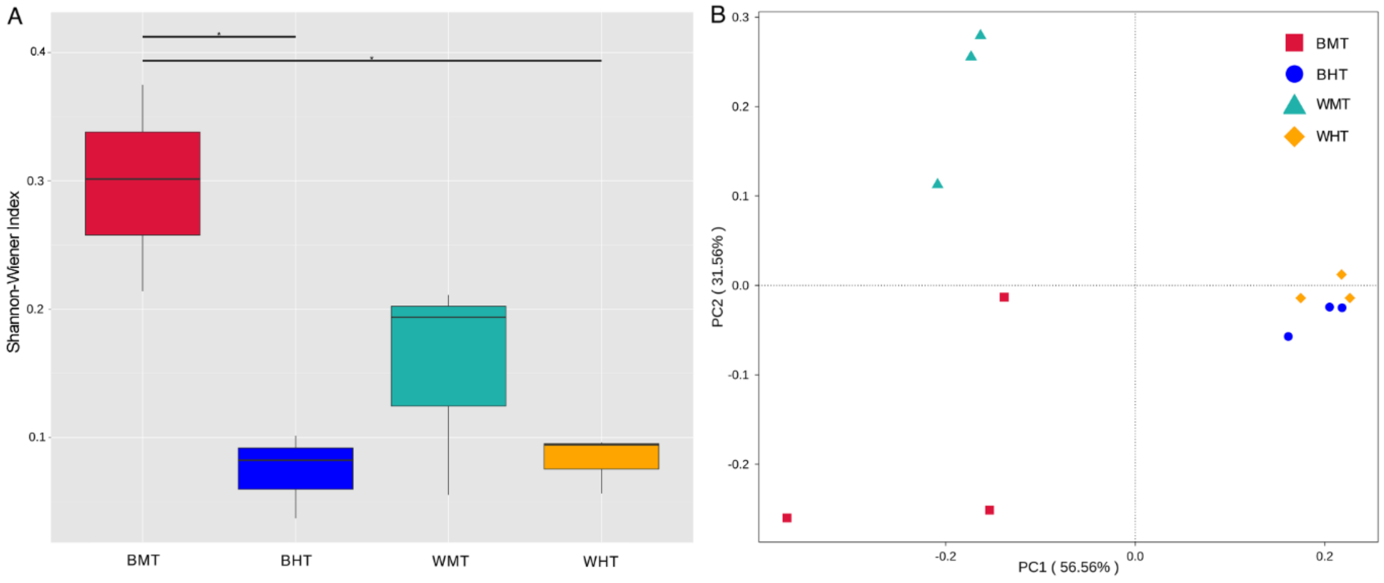

Table S1 Microbial samples used for this study.

Samples	Microbial samples of guts and food		Microbial cDNA samples of guts	
	Abbreviations	Numbers	Abbreviations	Numbers
Bamboo-feeding midgut	BM	3	BMT	3
Bamboo-feeding hindgut	BH	3	BHT	3
Bamboo fiber	BF	3	-	-
Wood-feeding midgut	WM	3	WMT	3
Wood-feeding hindgut	WH	3	WHT	3
Wood fiber	WF	3	-	-
In total		18		12

Table S2 One-way ANOVA analysis of alpha diversity of gut microbiome based on 16S rRNA gene.

Groups	ACE	Chao1	Shannon-Wiener	Simpson
BM	2546.15±144.41b	2709.65±182.22b	7.71±0.20b	0.9758±0.002b
BH	2428.14±109.63b	2648.64±93.96b	7.77±0.38b	0.9807±0.005b
BF	3310.87±568.20a	4269.68±1778.10a	8.80±0.52a	0.9917±0.004a
WM	2731.16±250.21b	3102.36±166.79ab	7.44±0.46b	0.9744±0.009b
WH	2345.28±273.81b	2515.02±279.93b	8.48±0.21a	0.9887±0.020ab
MF	3550.75±209.43a	3869.76±272.68ab	9.00±0.17a	0.9838±0.003a

Note: microbial DNA samples of guts and food: BM, bamboo-feeding midgut; BH, bamboo-feeding hindgut; BF, bamboo fiber; WM, wood-feeding midgut; WH, wood-feeding hindgut; WF, wood fiber.

Table S3 Pairwise analysis by Anosim based on Bray_Curtis distance of 16S rRNA gene.

Groups	Df	SumsOfSqs	MeanSqs	F.Model	R	*P*
BH vs WF	1(4)	1.174	1.174	17.971	0.818	0.001
BH vs WH	1(4)	0.329	0.329	14.023	0.778	0.1
BH vs BM	1(4)	0.475	0.475	22.305	0.848	0.001
BH vs WM	1(4)	0.693	0.693	16.060	0.801	0.001
BH vs BF	1(4)	1.185	1.185	14.408	0.783	0.1
WF vs WH	1(4)	1.239	1.239	21.539	0.843	0.1
WF vs BM	1(4)	1.129	1.129	20.394	0.836	0.1
WF vs WM	1(4)	0.870	0.869	11.258	0.738	0.001
WF vs BF	1(4)	0.329	0.329	2.8327	0.415	0.001
WH vs BM	1(4)	0.532	0.532	39.362	0.908	0.1
WH vs WM	1(4)	0.839	0.839	23.718	0.856	0.001
WH vs BF	1(4)	1.245	1.245	16.715	0.807	0.001
BM vs WM	1(4)	0.293	0.293	8.8332	0.688	0.001
BM vs BF	1(4)	1.109	1.109	15.34	0.793	0.1
WM vs BF	1(4)	1.030	1.030	10.935	0.732	0.1

Note: Df: degrees of freedom; SumsOfSqs: total variance; MeanSqs: mean square; F. model: F test value; R: sample difference degree of different groups (variance / total variance); the larger R, the higher the explanation difference; P value (< 0.05 means significant differences). Microbial DNA samples of guts and food: BM, bamboo-feeding midgut; BH, bamboo-feeding hindgut; BF, bamboo fiber; WM, wood-feeding midgut; WH, wood-feeding hindgut; WF, wood fiber.

Table S4 One-way ANOVA analysis of alpha diversity of gut microbiome based on cDNA of 16S rRNA gene.

Groups	ACE	Chao1	Shannon-Wiener	Simpson
BMT	1490.45±290.25ab	0.87±0.06b	1191.33±240.56a	5.50±1.19b
BHT	1585.13±23.76a	0.98±0.00a	1455.67±18.58a	8.16±0.03a
WMT	1193.39±43.81b	0.95±0.01a	914.00±60.89b	5.61±0.23b
WHT	1489.97±118.88ab	0.99±0.00a	1367.67±135.12a	8.16±0.28a

Note: microbial cDNA samples of guts: BMT, bamboo-feeding midgut; WMT, wood-feeding midgut; BHT, bamboo-feeding hindgut; WHT, wood-feeding hindgut.

Table S5 Pairwise analysis by Anosim based on Bray_Curtis distance of cDNA samples of 16S rRNA gene.

Groups	Df	SumsOfSqs	MeanSqs	F.Model	R	*P*
BHT vs WHT	1(4)	0.319	0.319	28.303	0.876	0.001
BHT vs WMT	1(4)	0.945	0.945	42.398	0.914	0.001
BHT vs BMT	1(4)	0.586	0.586	14.451	0.783	0.001
WHT vs WMT	1(4)	0.999	0.999	37.139	0.903	0.1
WHT vs BMT	1(4)	0.667	0.667	14.784	0.787	0.1
WMT vs BMT	1(4)	0.729	0.729	12.974	0.764	0.1

Note: Df: degrees of freedom; SumsOfSqs: total variance; MeanSqs: mean square; F.model: F test value; R: sample difference degree of different groups (variance/total variance), the larger R, the higher the explanation difference; *P* value: $P < 0.05$ means significant differences. Microbial cDNA samples of guts: BMT, bamboo-feeding midgut; WMT, wood-feeding midgut; BHT, bamboo-feeding hindgut; WHT, wood-feeding hindgut.

Table S6 The 31 lignocellulolytic bacteria isolates identified at genus level. Those thirteen isolates in bold showed higher cellulolytic efficiency in each genus and were assessed in bamboo fiber degradation.

	Anoxybacillus	*Bacillus*	*Enterobacter*	*Klebsiella*	*Pandoraea*	*Pseudomonas*	*Thermoflavimicrobium*
Midgut			**A1B**<br>5_A<br>AZA_4_1<br>AZA_4_2<br>AZA_4_3<br>AZA_4_4<br>**AZA_4_5**	EZ_3_1<br>EZ_3_2<br>**EZ_3_3**<br>**EZ_3_4**	**7_B**		
Hindgut	**H4A**	**D2A**	D4A<br>3_A<br>**DH_8_1**<br>DH_8_3<br>**DH_8_4**<br>DH_8_5	1_A<br>**4_A**<br>6_A<br>D3A<br>**AH_2_1**<br>AH_2_3<br>AH_2_4<br>AH_2_5		DH_1_A1<br>**DH_1_A3**	**1A**

Table S7 The natural bacterial system mixed according to the relative abundance of each isolate, showing in five mL culture.

Taxonomy	Isolates	μL in 5 mL	Sequence number of the most similar OTU in each gut sample												
			Mean	BM1	BM2	BM3	BH1	BH2	BH3	WM1	WM2	WM3	WH1	WH2	WH3
Anoxybacillus	H4A	250	504	280	402	680	120	226	185	2280	440	632	312	160	330
Bacillus	D2A	150	296	150	130	253	350	331	248	390	440	270	234	460	290
Enterobacter	A1B	500	996	500	592	599	1002	1079	1999	1520	1300	990	933	745	688
Enterobacter	AZA_4_5	500	1046	600	599	788	1200	1312	1432	1551	1600	1421	998	699	356
Enterobacter	DH_8_1	500	1005	754	335	699	1358	1224	1524	1654	1990	1321	662	331	211
Enterobacter	DH_8_4	500	1027	478	566	456	3211	2111	2345	2341	511	223	31	36	12
Klebsiella	EZ3_3	250	498	267	234	322	221	279	331	790	578	899	467	600	992
Klebsiella	EZ3_4	250	498	278	345	433	112	160	345	567	832	665	345	998	899
Klebsiella	4_A	250	499	299	322	456	123	975	334	321	567	559	970	77	990
Klebsiella	AH__1	250	504	556	66	346	779	663	234	557	890	432	500	521	498
Pandoraea	7_B	525	1047	600	345	552	542	552	2331	2221	234	453	234	2226	2276
Pseudomonas	DH_1_A3	600	1200	1139	1239	2288	468	442	456	3445	3214	683	562	256	213
Thermoflavimicrobium	1A	475	953	700	350	346	705	800	870	991	970	800	1002	1900	1996

Table S8 One-way ANOVA analysis of lignocellulose degradation efficiency.

Bacteria	0 h	12 h	24 h	2 d	3 d	5 d	10 d	15 d
AZA_4_5	5.00A	4.45±0.05a/B	4.30±0.96a/BC	4.15±0.13a/C	3.87±0.06a/D	3.70±0.10a/DE	3.53±0.06a/E	3.37±0.04a/E
DH_1_A3	5.00A	4.63±0.06b/B	4.55±0.05b/BC	4.43±0.03b/C	4.25±0.02c/D	4.14±0.01b/D	3.91±0.04b/E	3.60±0.06b/F
AVER	5.00A	4.63±0.06b/B	4.45±0.05b/C	4.25±0.05a/D	4.12±0.03b/D	3.95±0.04b/E	3.70±0.10c/F	3.52±0.03b/G
ABUN	5.00A	4.54±0.05ab/B	4.33±0.06a/BC	4.25±0.05a/C	4.08±0.03b/C	3.85±0.18a/D	3.41±0.03a/E	3.34±0.04a/E

Note: AVER, the artificial bacterial system prepared with equal proportions of each isolate; ABUN, the natural bacterial system mixed according to the relative abundance of each isolate, that revealed in the high-throughput sequencing results; AZA_4_5 and DH_1_A3, single bacterial strains. Lower letters indicate the significant differences among the bacteria groups in the same time period, while the upper letters indicating the significant differences among the time periods of the same bacteria group.
